# An Experimental Study on Partial Texture Reversibility After Accumulative Roll Bonding of a Cube-Oriented Aluminum Single Crystal

**DOI:** 10.3390/ma19153208

**Published:** 2026-07-27

**Authors:** Hui Wang, Junyao Dong, Rui Wang, Lihong Su, Yifan Wang, Shunjie Yao, Zhilan Ju

**Affiliations:** 1 School of Mechanical Engineering, Nantong University, Nantong 226019, China; hw737ntu@163.com (H.W.);; 2 Institute for Industrial Science, The University of Tokyo, Kashiwa 277-0882, Chiba, Japan; 3 School of Mechanical, Materials, Mechatronic and Biomedical Engineering, University of Wollongong, Wollongong, NSW 2522, Australia

**Keywords:** accumulative roll bonding, texture reversal, single crystal, slip activity

## Abstract

In this study, the through-thickness texture of an accumulative roll-bonding (ARB)-processed aluminum single crystal was characterized using the Electron Backscatter Diffraction (EBSD) technique, and the texture reversal during ARB was investigated. Partitioned crystal rotation about the transverse direction (TD) and the activation of two sets of slip systems that resulted in TD-rotation and slip traces were evidenced. Reduced TD-rotation in certain layers of 2-ARB and 3-ARB corresponded to the destruction of previously formed slip traces, which resulted from the activation of the other set of slip systems. The partial texture reversal in ARB was experimentally revealed for the first time, and the revealing of texture evolution mechanisms helps with texture tailoring for better formability of ARB-processed sheet metals.

## 1. Introduction

Among various severe plastic deformation (SPD) techniques, accumulative roll bonding (ARB) has the capability to fabricate bulk ultrafine-grained materials and hence has attracted wide research interest [1,2,3]. In the ARB process, the high friction between rolls and metallic sheets results in large gradients of shear strain through the thickness [4]. Due to the repeated cutting–stacking in the following cycles, the severely sheared surface is moved to the centre and undergoes compression deformation, and then moves towards the surfaces and experiences a combination of shear and compression deformation [5,6]. The transition between shear and compression deformation is a characteristic phenomenon of the ARB process [4,7].

The through-thickness shear strain has been experimentally measured in 1-ARB [8,9] and numerically predicted up to 3-ARB using the finite element method (FEM) [10]. The imposed shear strain plays a crucial role in determining texture evolution [11]. After one cycle, the large surficial shear strain results in a shear-type texture, and the low shear strain produces a rolling-type texture at the centre [12]. A textural transition from shear-type to rolling-type textures has been extensively characterized using experimental techniques or numerical methods [8,11,12], for which the change from shear deformation to compression was believed to be the reason. A further study revealed that the rate of textural transition is related to the magnitude of change in thickness position [13], i.e., thickness position-dependent.

In addition to the change in non-uniform through-thickness strain, shear strain reversal in certain layers has been analytically calculated [13] and numerically predicted using FEM [6], which is called partial shear strain reversal. This partial reversal of shear strain is due to the cutting–stacking pattern of ARB [13], not caused by changing rolling direction. The partial texture reversal could be another potential reason for the relatively lower ARB texture intensity compared to conventional rolling [11,12]. However, the locations and magnitude of texture reversal have not been thoroughly investigated, and this is the purpose of the present study.

In this study, an aluminum single crystal was chosen, since its known initial orientation enabled the tracing of texture evolution. After ARB processing, the through-thickness texture was characterized using the electron backscatter diffraction (EBSD) technique. The texture evolution was traced up to 3 ARB cycles, and partial texture reversal has been experimentally observed for the first time. The texture reversal and alternation of slip activities in this study and previously published studies were investigated and discussed.

## 2. Materials and Experiments

The material used in this study was pure aluminum single crystal (purity ≥ 99.99%), which was supplied by MTI Corporation, Richmond, CA, USA (https://mtixtl.com/en-euea) (accessed on 15 April 2024). The sample dimensions were 50 × 10 × 1 mm^3^ (length × width × thickness). The initial orientation was Cube {0 0 1} <1 0 0>, (0 0 1) plane normal oriented parallel to ND and [1 0 0] crystal directions oriented along RD. The starting orientation was measured using XRD, and the initial orientation was accurate within a tolerance of 2° from the ideal Cube.

**Figure 1:**
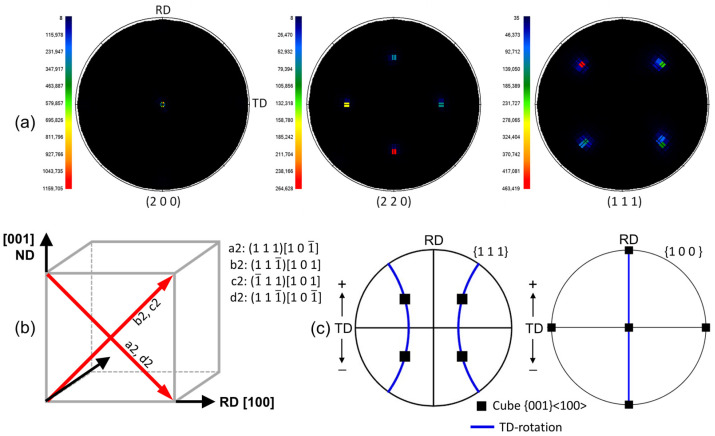
(**a**) Three pole figures show the accuracy of the initial orientation. (**b**) Relation between the sample coordinates (RD, TD, ND) and the crystal coordinates of the Cube {0 0 1} <1 0 0>-oriented aluminum single crystal, and two sets of slip systems, a2-d2 and b2-c2, having the highest Schmid factors. (**c**) {1 1 1} and {1 0 0} pole figures show the position of the Cube {0 0 1} <1 0 0> orientation and partitioned crystal rotation about TD.

The relationship between the sample coordinate and crystal coordinate is shown in Figure 1b. Table 1 lists the notation of the 12 {1 1 1} <1 1 0> slip systems in FCC-structured aluminum, and the corresponding Schmid factor. When calculating the Schmid factor, a triaxial stress system is usually assumed for rolling deformation [14]. In the triaxial stress system, a stress σ is parallel to the ND, −nσ parallel to the RD, and 0.5∗(σ−nσ) parallel to the TD, where n is a value in the range from 0.5 to 1. Under this stress state, two sets of slip systems, a2-d2 and b2-c2 (Figure 1b), possess the highest Schmid factor and accordingly are considered to be activated [14,15].

**Figure 2:**
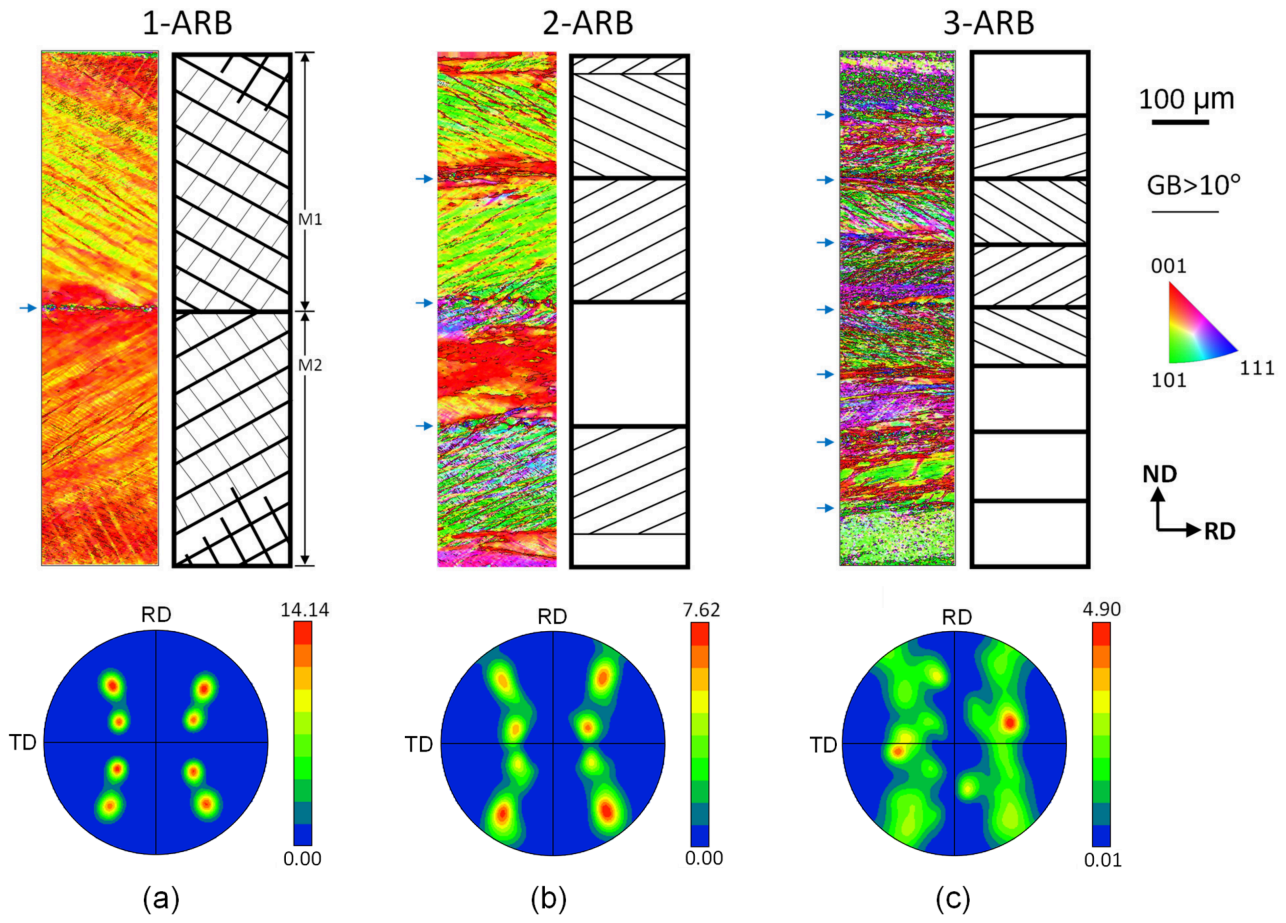
EBSD IPF maps, schematically presentation of recognized slip traces, and {1 1 1} pole figures after (**a**) 1-ARB, (**b**) 2-ARB, and (**c**) 3-ARB. The blue arrows indicate the bonded interfaces.

The identification of the activated slip systems is solely based on the Schmid factor, which reflects the geometric favourability of slip under the plane strain compression, since the Schmid factor was regarded as the first-order indicator. In contrast, the other factors (e.g., latent hardening, strain path, and local stress heterogeneity) played a secondary role in slip activities, especially in single crystals. The following EBSD maps in Figure 2 also clearly show this assumption. Additionally, the activated slip systems under this stress assumption have been extensively validated by experimental observations [14,15,16] and crystal plasticity modelling [17,18,19]. More comprehensive analyses (e.g., crystal plasticity modelling) would be required to quantify the effects of slip interactions and local stress evolution.

According to the method proposed in Ref. [16], the crystal rotation was partitioned into rotations about the three axes of sample coordinates, i.e., RD-rotation, TD-rotation, and ND-rotation. Specifically, the misorientation of a CPFEM element relative to the average crystal orientations was represented by a misorientation matrix Gac, Gac=(Gic)(Gai), where the ‘*c*’, ‘*a*’, and ‘*i*’ subscripts respectively stand for the crystal orientation, average orientation, and intermediate orientation. When calculating the Gac about a particular direction [*hkl*], Gai is the associated axis/angle [*hkl*]/α, and Gic is the one with [*mnp*]/β. For a crystal orientation expressed by Gac, and rotation axis defined as [*hkl*], the angle α can be found by minimizing β according to

(1) tanα=h(Gac)32−Gac23+k[Gac13−Gac31]+l[Gac)21−(Gac)12]{(h2−1)Gac11+(k2−1)Gac22+(l2−1)Gac33+hk[Gac12+Gac21]+kl[Gac23+Gac23]+lh[Gac31+Gac13]}

By using this method, the crystal rotation relative to the initial orientation was partitioned into three components (RD-, TD-, and ND-rotation). The method expressed by Equation (1) was useful when the crystal rotation was strongly dominant about one particular axis, since the primary rotation component (represented by Gac) is maximized by associating the Gai to the primary rotation axis. Among these three partitioned rotations, TD-rotation is dominant over RD-rotation and ND-rotation in Cube-oriented single crystals up to 3 cycles, and thus only the path of TD-rotation from the initial Cube is shown in Figure 1c.

In ARB processing, two sheets were stacked together after their contacting surfaces were wire-brushed, rolled with a nominal reduction of ~50%, and then cut into two halves. The sheets were then cleaned with acetone, wire-brushed, and stacked before being rolled again. After rolling, the sheets were cooled in water immediately. The ARB experiment was repeated up to 3 cycles, since the crystal rotation about the TD is dominant within 3 cycles. The rolls were 125 mm in diameter, and the rolling speed was 196 mm/s. The ARB experiment was conducted without lubricant, and the roll surface was cleaned with acetone before rolling. The ARB process was performed at room temperature. Four batches of ARB experiments were performed, and one batch failed at achieving good bonding. The EBSD results of these three batches of samples were similar after 3-ARB, and thus one representative experiment batch was chosen for EBSD mapping.

After rolling, the through-thickness texture was characterized by EBSD on a JEOL JSM-7001F (JEOL Ltd., Tokyo, Japan). EBSD samples were taken from the RD-ND cross-section located at the centre of the rolled sheet after each ARB cycle. This EBSD scanned area was taken from the steady-rolling region, and the EBSD observation revealed that the textures of these three batches of ARB-processed samples were similar. Before EBSD characterization, the samples were ground and electrolyte-polished. The EBSD observed areas were extended as close to the surfaces as possible, i.e., ~1000 μm along ND and 200 μm along RD, and a step size of 1 μm was used. The EBSD mapping was performed with an accelerating voltage of 15 kV and a working distance of 15 mm.

Channel-5 was adopted for post-processing the EBSD data. When performing the clean-up procedure, misorientation angles below 2° were disregarded, and subgrain/grain smaller than 3 pixels were neglected. The three Euler angles of every EBSD point were used to calculate the TD-rotation. The Euler angles were converted to the Miller indices, and then TD-rotation was calculated according to Equation (1) [16]. For the TD-rotation angle at a particular thickness position, its value was averaged from all the EBSD points at the same thickness position.

## 3. Results

**Figure 3:**
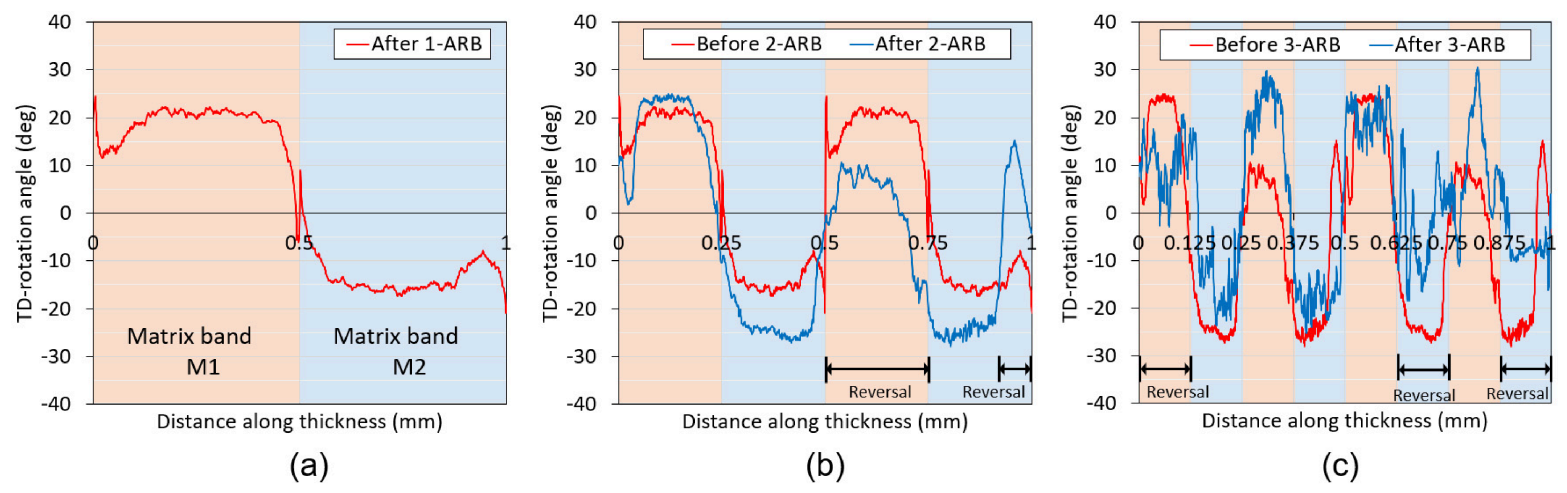
Partitioned TD-rotation angles along the whole thickness (**a**) after 1-ARB, (**b**) before and after 2-ARB, and (**c**) before and after 3-ARB.

Figure 2 shows the EBSD maps and {1 1 1} pole figures, and Figure 3 shows the partitioned TD-rotation calculated from the EBSD results. There are 2, 4, and 8 stacked layers after 1-ARB, 2-ARB, and 3-ARB, respectively. It needs to be noted that the bonded interface is around tens of microns (Figure 2a) and therefore the effect of wire brushing can be reasonably discarded.

### 3.1. 1-ARB

The EBSD inverse pole figure (IPF) maps in Figure 2a clearly show the slip traces after 1-ARB, as schematically shown in Figure 2a. According to the direction of slip traces, the whole thickness is divided into two matrix (M) bands, M1 and M2. The slip traces in M1 and M2 are due to the activation of the a2-d2 set and b2-c2 set of slip systems, respectively (Figure 1b). The slip traces are at an intersecting angle of about $\pm 40^{{}^{\circ}}$ to RD, but they change direction at the bonded interface. The activation of the two sets of slip systems resulted in TD-rotation (Figure 2a and Figure 3a). The TD-rotation is positive in M1, while negative in M2. A ‘+ −’ pattern of TD-rotation developed through the thickness. TD-rotation is exceedingly larger than RD- and ND-rotation, as indicated by the {1 1 1} pole figure in Figure 2a and the IPF colours in Figure 2a.

### 3.2. 2-ARB

After 2-ARB, TD-rotation is still strongly dominant over RD-rotation and ND-rotation, according to the {1 1 1} pole figure and the IPF colours in Figure 2b. The slip traces can be clearly seen in the first, second, and fourth layers (Figure 2b), which means one set of slip systems is dominant over the other sets. In contrast, the slip traces are not evident in the third layer, which means the slip traces formed in 1-ARB were destroyed in 2-ARB. This destruction implies that the b2-c2 set of slip systems other than the a2-d2 set in 1-ARB was activated in 2-ARB.

At the beginning of 2-ARB, two 1-ARB processed sheets were stacked, and accordingly, a “+ − + −” pattern of TD-rotation is seen along the whole thickness before 2-ARB (Figure 3b). Compared to that before 2-ARB, the TD-rotation obviously increases in the first, second, and fourth layers, which is consistent with the intensified slip traces in these corresponding layers. In contrast, the TD-rotation is surprisingly and exceedingly decreased in the third layer of 2-ARB. This means the TD-rotation is clockwise in 1-ARB, but anticlockwise in 2-ARB. The reduced TD-rotation corresponds to the destruction of previously developed slip traces in the third layer of 2-ARB (Figure 2b). This reduced crystal rotation only occurred in the third layer out of the total four layers, which is called partial texture reversal in this report, as these layers/regions were marked by ‘reversal’ in Figure 3b. This unusual phenomenon in ARB has not been experimentally revealed before.

### 3.3. 3-ARB

Eight layers were stacked along the thickness in 3-ARB. Slip traces were intensified in the second, third, fourth, and fifth layers, while the two sets of slip traces were almost equivalent in the other layers (Figure 2c). The crystal rotation angles also increased after 3-ARB (Figure 2c). TD-rotation still dominated over RD-rotation and ND-rotation, though RD-rotation and ND-rotation started to evolve (Figure 2c). The comparison of TD-rotation between before 3-ARB and after 3-ARB clearly shows that the TD-rotation increases in the third layer and is almost maintained in the second, fourth, and fifth layers, and this corresponds to the intensified or maintained slip traces in these layers. The reduced TD-rotation in the other layers (first, sixth, seventh, and eighth layers) is consistent with the destroyed slip traces. Overall, partial texture reversal also occurred in 3-ARB, and these regions were also marked by ‘reversal’ in Figure 3c.

## 4. Discussion

### 4.1. Validation of Experimental Observations

The rolling process could be influenced by the sample thickness, sample surface treatment, lubrication conditions, rolling temperature and speed, etc., and thus the ARB process in each cycle is not exactly the same. Considering these factors influencing rolling conditions, the authors believe that the experimentally observed partial texture reversal in this study is not an occasional phenomenon, since the regions of texture reversal are obviously large and observed in both 2-ARB and 3-ARB (Figure 3).

**Figure 4:**
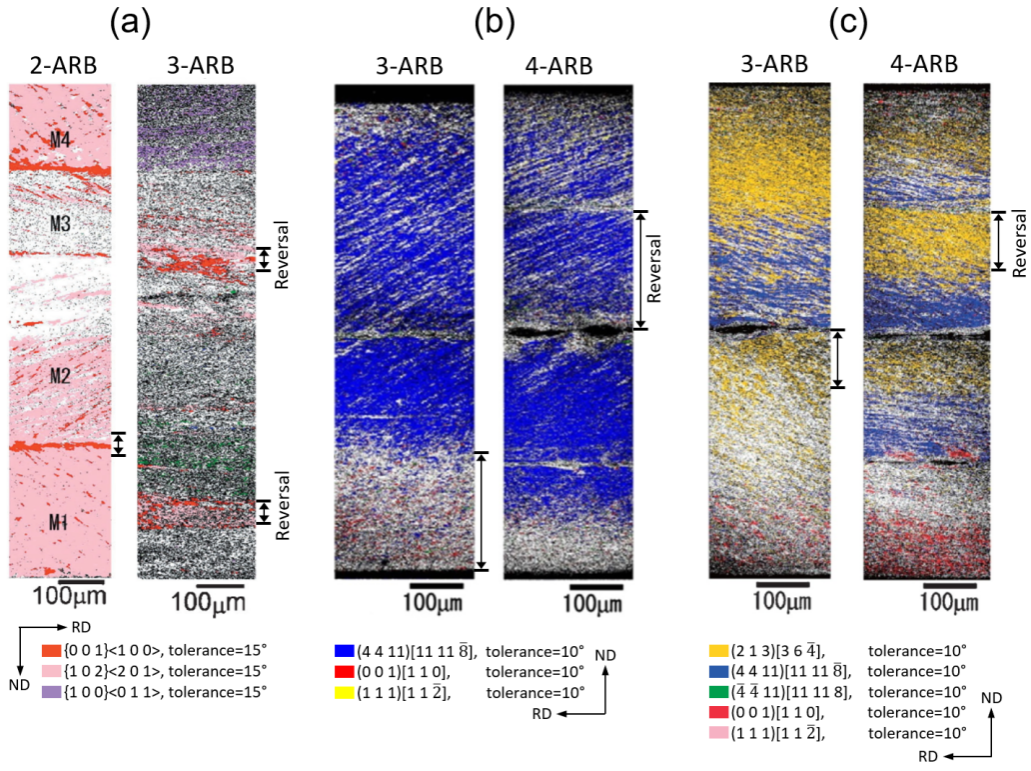
Texture components in ARB-processed aluminum single crystals with orientations of (**a**) {1 0 0} <0 0 1> [20], (**b**) {1 1 2} <1 1 1> [21], and (**c**) {2 1 3} <6 3 4> [22]. The regions of partial texture reversal are marked.

Additionally, a re-analysis revealed that partial texture reversal occurred in all previously published ARB-processed aluminum single crystals [20,21,22]. Figure 4 summarizes the EBSD observations of these three ARB-processed single crystals, and the regions of texture reversal in the previous and next cycles are indicated by ‘Reversal’. The aluminum single crystals were rolled at room temperature with lubrication, and the roll diameter was 310 mm. The 4 mm thick sheet was rolled to 2 mm by conventional rolling (without cutting–stacking and roll-bonding), and the typical ARB process was used in the following cycles. After the ARB process, the through-thickness texture on the RD-ND plane was characterized using EBSD, and the EBSD step size was 1 µm/0.5 µm. A tolerance of 10°/15° was used to identify the texture components. For instance, the initial orientation of the aluminum single crystal in Figure 4c was {2 1 3} <3 6 4>, and the crystal orientations rotated away from the initial {2 1 3} <3 6 4> with increasing strain. The fraction of {2 1 3} <3 6 4> in the marked region of 3-ARB (Figure 4c) is very low, but the fraction increased significantly in this region after 4-ARB. This is to say that the starting texture {2 1 3} <3 6 4> was destroyed by crystal rotation at first, and then the initial {2 1 3} <3 6 4> was recovered by crystal rotation toward the {2 1 3} <3 6 4>. This is texture reversal and partially occurred in the marked region. Similarly, the partial texture reversal of the initial orientations, {1 0 0} <0 0 1> and {1 1 2} <1 1 1>, also occurred in Figure 4a and Figure 4b, respectively. In Figure 4, the re-analysis of texture reversal is concerned with the starting orientations, since only the starting orientations can be clearly identified according to the published EBSD maps. In contrast, the crystal rotation angles were used to show the texture reversal in the current report.

### 4.2. Partial Texture Reversal in ARB

Multi-pass unidirectional and reverse rolling of aluminum single crystals {0 0 1} <1 0 0> has been conducted, and the through-thickness crystal rotation has been experimentally observed using the EBSD. After multi-pass unidirectional rolling, the previously developed through-thickness patterns characterized by TD-rotation and slip traces were amplified, since the same through-thickness shear strain was imposed and hence the same set of slip systems was primarily activated. In contrast, the previously developed TD-rotation and slip traces were destroyed after multi-pass reverse rolling, where the sheet was fed by rotating about the ND for $180^{{}^{\circ}}$ after each pass. In multi-pass reverse rolling, the imposed shear strain was in the opposite direction after each pass, and the primary and secondary sets of slip systems alternated after each pass. This resulted in destroyed slip traces and reduced TD-rotation that previously developed in the last pass.

In multiple passes of processing (e.g., multi-pass rolling, ARB, equal channel angular pressing), the final developed features are the competition results between previously developed features (e.g., microstructure and texture) and newly imposed strain [8,23]. In multi-pass unidirectional rolling, the newly imposed deformation is consistent with the previously developed features, and hence the previously formed features are amplified. This also explains the destruction of previously evolved features in multi-pass reverse rolling. The amplification and destruction of previously developed deformation behaviours in the unidirectional rolling and reverse rolling, respectively, have also been successfully reproduced by crystal plasticity FEM (CPFEM) [17]. The amplification and destruction of deformation features in unidirectional rolling and reverse rolling, respectively, almost occurred along the whole thickness [15]. The textural reversal and alternation of slip traces in ARB are partial, not along the whole thickness. The partial texture reversal is consistent with the change in shear strain direction in these regions, as the shear strain revealed by FEM [6] and CPFEM [17,18]. The plastic deformation and texture evolution are fully coupled in metal processing. Texture influences the distribution of introduced strain, and hence the imposed shear strain is different from cycle to cycle. This explains why the positions of texture reversal vary between cycles (Figure 3).

In ARB, it is shear and compression deformation at the surfaces and centre, respectively [9]. The shear strain results in shear-type texture (typical includes {0 0 1} <1 1 0>, {1 1 1} <1 1 2>, and {1 1 1} <1 1 0>), while the compression deformation leads to a rolling-type texture (consisting of {1 1 2} <1 1 1>, {1 2 3} <6 3 4>, and {1 1 0} <1 1 2>). The highly shear-deformed surface in the previous ARB cycle is moved to the centre in the next cycle, and this through-thickness position movement is accompanied by the textural transition from shear-type to rolling-type texture [7]. In the following cycles, the centre is gradually relocated towards the surfaces. This shear-compression deformation transition is believed to be the reason for textural transition, which has been widely observed in ARB-processed polycrystalline metals [7,24].

Cube-oriented single crystals were used in this report to reveal the partial texture reversal. Additionally, the partial texture reversal can also be seen from all three published ARB-processed aluminum single crystals according to our re-analysis. The ARB experiment in this study was conducted up to 3 ARB cycles, since non-TD-rotation started to develop after 3-ARB and the development of non-TD-rotation led to poor comparison in crystal rotation between before and after an ARB cycle. The texture reversal is also expected after 3-ARB, since a gradual change of the single crystals to polycrystals with increasing strain and textural reversal has also been widely observed in ARB-processed polycrystals. Partial texture reversal has also been reported according to experimental characterization [13] and crystal plasticity finite element (CPFEM) modelling [18]. It is also claimed that the shear strain reversal was the reason for the partial texture reversal.

In FCC-structured metals (e.g., aluminum in this study), ARB results in a rolling-type texture, and the undesired texture in the as-rolled metallic sheets usually leads to poor formability [15,25]. Understanding the mechanisms of texture evolution is crucial to tailoring texture [7,24]. The current study demonstrates that partial texture reversal occurs in conventional ARB, i.e., without changing the rolling direction. The partial reversal of texture and shear strain is due to the cutting–stacking in ARB. This partial reversal of shear strain is believed to be another reason for the low texture intensity and texture transition between the shear-type and rolling-type texture in ARB-processed metals. Based on the findings of partial texture reversal in conventional ARB, modifying ARB processes and adjusting ARB parameters can be used to tune the texture in ARB-processed sheet metals [24,26].

## 5. Conclusions

1. A Cube-oriented aluminum single crystal was processed by ARB up to 3 cycles, and through-thickness crystal rotation angles and slip traces were characterized using the EBSD technique.
2. The activities of two slip system sets represented by slip traces and partitioned TD-rotation were correlated.
3. For the first time, partial texture reversal was clearly observed in 2-ARB and 3-ARB, and the texture reversal corresponded to destroyed slip traces due to the activation of the other set of slip systems.
4. Our re-analysis suggests that partial texture reversal was also presented in all reported ARB-processed aluminum single crystals.
5. This study experimentally revealed partial texture reversal in conventional ARB, and the findings shed light on texture tailoring in ARB-processed sheet metals.

## Figures and Tables

**Table 1:** Notation of 12 slip systems and corresponding Schmid factor.

Slip plane	(1 1 1)			($\bar{1}$ $\bar{1}$ 1)			($\bar{1}$ 1 1)			(1 $\bar{1}$ 1)		
Slip direction	[0 1 $\bar{1}$]	[$\bar{1}$ 0 1]	[1 $\bar{1}$ 0]	[0 $\bar{1}$ $\bar{1}$]	[1 0 1]	[$\bar{1}$ 1 0]	[0 1 $\bar{1}$]	[$\bar{1}$ $\bar{1}$ 0]	[1 0 1]	[0 $\bar{1}$ $\bar{1}$]	[$\bar{1}$ 0 1]	[1 1 0]
Slip system	a1	a2	a3	b1	b2	b3	c1	c3	c2	d1	d2	d3
Schmid factor	0.2	−0.41	0.2	0.2	−0.41	0.2	0.2	−0.41	0.2	0.2	−0.41	0.2

## Data Availability

The original contributions presented in this study are included in the article. Further inquiries can be directed to the corresponding authors.
